# Study on Dynamic Mechanical Properties of Sandwich Beam with Stepwise Gradient Polymethacrylimide (PMI) Foam Core under Low-Velocity Impact

**DOI:** 10.3390/ma17092099

**Published:** 2024-04-29

**Authors:** Mousab Mahgoub, Cong Liu, Zhuhua Tan

**Affiliations:** School of Mechanical Engineering, Hebei University of Technology, Tianjin 300401, China; mousaba71@gmail.com (M.M.);

**Keywords:** stepwise gradient, sandwich structure, low-velocity impact, failure mode, energy absorption

## Abstract

Different PMI foam materials of 52, 110, and 200 kg/m^3^ were used to design stepwise gradient cores to improve the impact resistance of the sandwich beam. The stepwise gradient core consists of three layers arranged in positive gradient, negative gradient, and sandwich-core (e.g., 200/52/200). These sandwich beams were subjected to the impact of a steel projectile under impact momentum of 10 to 20 kg·m/s, corresponding to impact energy in the range of 12.5 to 50 J. During the test, the impact force was recorded by an accelerometer, and the different failure modes were also obtained. Subsequently, the influence of the layer arrangement on the energy absorption and load transfer mechanism between the different layers was analyzed. The results showed that the top layer with a large density can improve the impact force, but the middle/bottom layer with a low density promoted specific energy absorption. Thus, based on these two points, the negative gradient core (200/110/52) had an excellent specific energy absorption because it can transfer and expand the area to bear the load layer by layer, which improved the energy absorption in each layer. Combined with the failure modes, the load transfer and deformation mechanisms between the layers were also discussed. The present work provided a valuable method to design an efficient lightweight sandwich structure in the protection field.

## 1. Introduction

The sandwich structure consists of two face sheets and a core, which has been widely used in the fields of aerospace, automotive, marine vehicle, and other industry applications due to their attractive properties, such as high specific strength, high specific stiffness, and excellent energy absorption [1,2,3,4,5,6].

Over the past decades, a great deal of work has been conducted on the quasi-static and dynamic behavior of the different sandwich structures (e.g., beams [7], plates [8], shells [9], and tubes [10]), and the corresponding failure modes and mechanisms were extensively studied, such as indentation [11], core shear failure [12], tension failure [13], and debonding [14]. It is well known that the core is a key component of the sandwich structure, which not only suffers from the shear load but also dissipates energy through deformation or collapse failure. Different lightweight materials and structures have been used to design the core, such as aluminum foam [15], polymeric foam [16], honeycomb core [17], lattice core [18], corrugated core [19], etc. To some degree, most of these research studies focused on the core with uniform or monolithic materials or structures. Thus, the development of an efficient core is still a challenge in the field of sandwich structures.

Recently, the primary concept of density-graded foams was developed to improve the load-bearing capacity and energy absorption of the sandwich structures, which consisted of several layers with different densities. Lots of work has been conducted on the compressive mechanical properties of density-graded foam under quasi-static and dynamic loading [20,21,22,23,24], such as deformation and failure mechanisms [20], stress wave propagation [21], stress–strain response [22], constitutive model relationship [23], and energy absorption characteristics [24]. These works revealed a noticeable difference in the deformation mechanism between quasi-static and dynamic loading due to the inertia efficiency of the impact loading. Moreover, the arrangement of the density-graded foam layers significantly influenced deformation and energy absorption [20,21,24,25,26,27,28,29,30,31,32,33,34,35]. Koohbor et al. [20] demonstrated that gradient greatly influences the propagation of stress waves in foams with density gradients. Furthermore, the overall ability of the structure to absorb energy corresponds to the stability of the shock waves through the gradient foam. Kazemi et al. [25,26] studied the sandwich beam/panel with stepwise density-graded cores under quasi-static three-point bending and punch loading. The results showed that the arrangement of the density layer and the number of layering have the most significant influences on the bending resistance, contact peak force, and energy absorption. Nie et al. [27] reported the effects of the density-graded changes of foam core sandwich panel subjected to high-velocity impact, and they concluded that the sandwich panel with a stepwise negative gradient core outperforms to resist the perforation and energy dissipation compared to a positive gradient and uniform-density core sandwich panel. In addition, the failure modes and deformation mechanisms of stepwise density gradient sandwich structures under different load cases were also investigated, such as flexural bending [28,29], blast loading [30,31], low-velocity impact [32,33], and high-velocity impact [34,35].

However, the choice of materials with high specific strength, stiffness, and excellent energy absorption is another significant way to improve the mechanical performance of the sandwich structure. Polymethacrylimide (PMI) material is lightweight and has high energy absorption, which has attracted extensive attention in recent years. The sandwich structure with PMI core has been applied in transportation, aerospace, and military protection equipment [36,37,38,39]. The mechanical properties of the sandwich structure with PMI core were also focused under different load cases, such as indentation load [36], three-point bending load [37], damage and fatigue [38], and energy absorption [39]. Most of these works focused on increasing the strength and stiffness of sandwich structures by increasing the density and thickness of the PMI core [40,41] and filling the honeycomb core with PMI foam materials [42].

Most of the previous work focused on sandwich structures with a single-layer PMI foam core, and the work on the sandwich beams with stepwise density-graded PMI foam core is seldom performed. Therefore, in the present paper, the PMI foam materials with different densities of 52, 110, and 200 kg/m^3^ were used to design the stepwise gradient core of the sandwich beam. The dynamic mechanical properties of the sandwich beam were studied under the impact of a steel projectile. The impact force was recorded using an accelerometer during the test, and different failure modes were obtained. Based on the experimental results, the effects of the arrangement of the layers on the energy absorption and specific energy absorption were analyzed. The corresponding deformation and failure mechanisms were also discussed.

## 2. Experimental Procedures

### 2.1. Materials and Specimens

**Figure 1:**
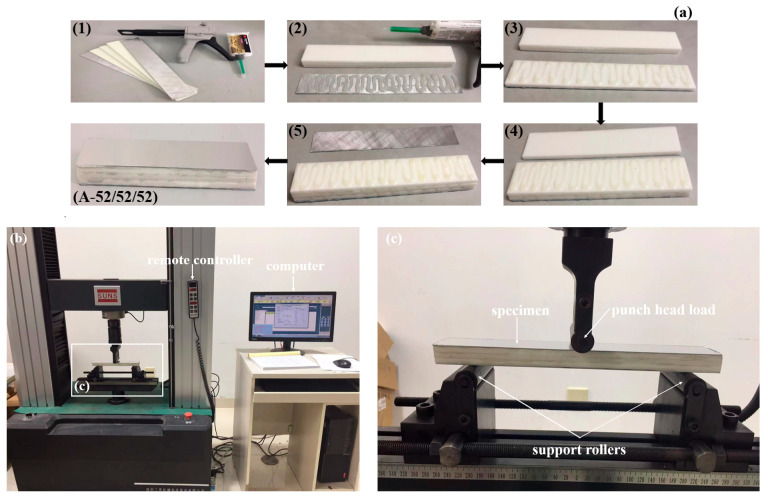
Specimen and the three-point bending test. (**a**) The fabrication steps of the sandwich beam with stepwise gradient core (step 1 is the cutting of the face sheets and foam core from the large plates with specific dimensions of the sandwich beam while steps 2–5 are the gluing the lower face sheet with the bottom layer foam core and consequentially gluing other layers core and upper face sheet); (**b**) the three-point bending test; (**c**) enlarged view of the specimen and support rollers.

PMI foam materials (purchased from Suzhou Zhongbao Composite Material Co., Ltd., Suzhou, China) [43] with different densities of 52, 110, and 200 kg/m^3^ were used to design a stepwise gradient core for the sandwich beam. Aluminum alloy AL-1050 (purchased from Lutai Co., Ltd., Suzhou, China) [44] was used to design the face sheet of the sandwich beam. The dimensions of the sandwich beam were 260 mm in length and 40 mm in width. The thickness of the face sheet was 0.5 mm. The core consisted of three layers of PMI foams with different densities of 52, 110, and 200 kg/m^3^, and each layer was 5 mm thick. The face sheets and PMI foam layers were glued together in different arrangements using epoxy resin (produced by 3M Scotch-Weld Co., Ltd., St. Paul, MN, USA), and the fabrication steps of the specimen are shown in Figure 1a. Five types of stepwise gradient cores were designed as follows: (1) sandwich-core 52/X/52, which means the top and bottom layers of the sandwich-core are 52 kg/m^3^ in density, and the middle layer varied from 52 kg/m^3^ to 200 kg/m^3^ and is denoted as X; (2) sandwich-core: 110/X/110; (3) sandwich-core: 200/X/200; (4) positive gradient core: 52/110/200; (5) negative gradient core: 200/110/50. More details are listed in Table 1.

### 2.2. Experimental Apparatus

The quasi-static three-point bending tests were performed by a universal material testing machine SUNS WAW000 (product of Shenzhen SUNS Technology Stock Co. Ltd., Shenzhen, China) according to the ASTM C-393 test standard [45], as shown in Figure 1b,c. Two rollers supported the specimen, and two ends of the specimen could not move in the vertical direction. The compression load was applied on the mid-span of the specimen by a steel compression head. During the tests, the friction force between the bottom face sheet of the sandwich beam and the two support rollers is little, which can be neglected [45]. The effective length was 200 mm between the two support rollers [46]. The load rate was 2 mm/min, and a computer was connected to the machine to record the load and displacement data.

**Figure 2:**
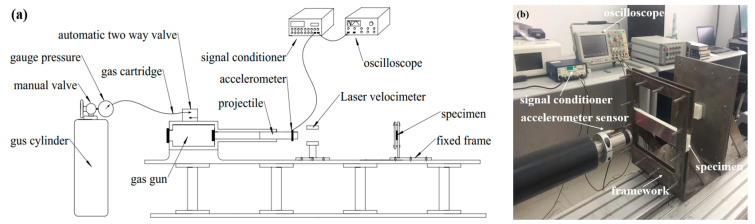
The impact apparatus for the dynamic experiments: (**a**) schematic graph; (**b**) experimental apparatus.

For the impact tests, a steel projectile was launched by a gas gun and impacted the clamped sandwich beam at the mid-span position, as shown in Figure 2. The mass of the steel striker bar was 8 kg, and the dimensions were 40 mm in diameter and 800 mm in length. Two ends of the sandwich beam were fixed in a frame, and the effective span was 200 mm. The projectile’s velocity was controlled by gas pressure and measured using a laser velocimeter. Moreover, a 352C04 PCB accelerometer (produced by PCB Company, Depew, NY, USA), with a sensitivity of 2.51 mV/g, was fixed on the projectile to measure the impact force, as shown in Figure 2b. The sampling rate was 5 MHz during the tests, and the accelerometer signal was amplified by a PCB 482C05 signal conditioner (produced by PCB Company, Depew, NY, USA) and saved in the Tektronix MDO3034 oscilloscope (produced by Tektronix, Beaverton, OR, USA).

## 3. Results and Discussions

### 3.1. Quasi-Static Load Case

#### 3.1.1. Compressive Force–Displacement Curves

**Figure 3:**
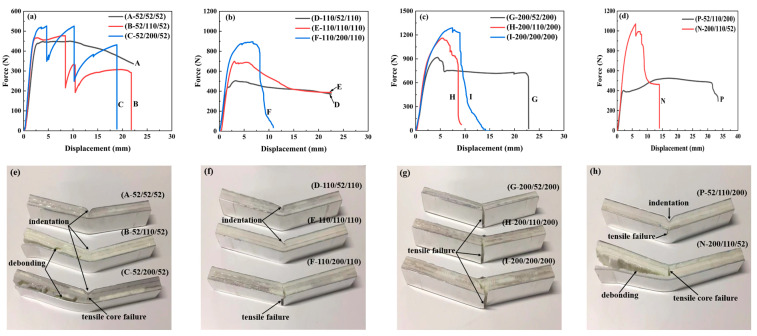
Force–displacement curves and failure modes of the sandwich beams with different cores under the three-point bending tests: (**a**,**e**) for 52/X/52; (**b**,**f**) for 110/X/110; (**c**,**g**) for 200/X/200; (**d**,**h**) for 52/110/200 and 200/110/52, respectively.

Figure 3 illustrates the sandwich beams’ force–displacement curves and failure modes with different cores in the three-point bending tests. It can be found that the arrangement of the layers in the core has a significant influence on the peak force. The peak force increased from 450 N to 1288 N for the different sandwich beams. Moreover, there is a sudden change in the curve of a sandwich beam of 52/110/52 and 52/200/52 in Figure 3a, and the rest of the sandwich beams had a similar tendency in the force–displacement curves.

For sandwich-core of 52/X/52, the top layer with low strength easily fails in indentation failure mode, and if the middle layer is also 52 kg/m^3^ in density, all the layers would fail in the same mode; if the middle layer is with a density of 110 or 200 kg/m^3^, the strength of the middle layer increased, which would improve the load bearing capacity and prevent the drop of the curves. This is also another reason for the sudden curve changes in Figure 3a.

For sandwich-cores of 110/X/110 and 200/X/200, the curves ascended as the density of the middle layer increased, as shown in Figure 3b. The bearing load is because the top and bottom layers have a larger strength and can suffer the external load, which determines and transfers the load to the following middle layer. Though the stiffness and strength increased, the deformation decreased, which is much less than those of 52/X/52.

For the positive gradient core, the arrangement of the layers is 52, 110, and 200 kg/m^3^ in the sequence. The top layer of 52 kg/m^3^ in density has a low strength, which would undergo a similar deformation process and failure mechanism of 52/X/52; the top layer would fail at the load exertion point, then the load keeps the concentration state, which cannot be expanded effectively. However, for the negative gradient core, on the contrary, the arrangement of the layers is 200, 110, and 52 kg/m^3^ in the sequence for the negative stepwise gradient core; the top layer of 200 kg/m^3^ has enough strength to suffer the external load and distribute the concentrated load, then the distributed load was transferred to the next layer. The area of load exertion on the following layers would expand layer by layer. Thus, the middle and bottom layers can sustain the load and absorb the energy efficiently.

#### 3.1.2. Energy Absorption of the Sandwich Beam under Three-Point Bending Tests

The energy absorption (*EA*) of the sandwich beam was calculated by using the force-displacement curve, and the specific energy absorption (*SEA*) was obtained by dividing the energy absorption by the average density of the core. The corresponding equations are as follows [40]:

(1) EA=∫Fdδ

(2) $$SEA=\frac{\int Fd\delta }{\bar{\rho }}$$

where F and δ are the corresponding force and displacement, EA represents energy absorption, SEA is the specific energy absorption, and $\bar{\rho }$ denotes the average density of the core.

**Figure 4:**
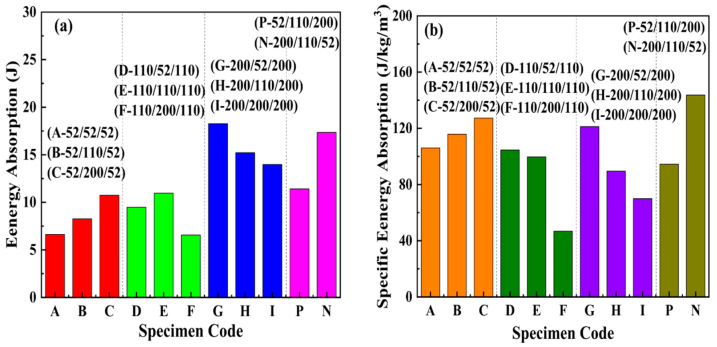
(**a**) Energy absorption and (**b**) specific energy absorption of the sandwich beam with different cores.

Figure 4 illustrates the energy absorption (*EA*) and specific energy absorption (*SEA*) of the sandwich beams with different cores. During the calculation of the *EA* and *SEA*, the influence of the face sheet on the EA and SEA was neglected. Because the thickness of the face sheet is just 0.5 mm, the stiffness and the corresponding load-bearing capacity are small. For the sandwich-core of 52/X/52, both EA and SEA of the sandwich beam increased with the increasing density in the middle layer. The increasing density in the middle layer can improve the stiffness of the sandwich beam, which can bear a larger load and absorb more energy. However, for the sandwich-cores of 110/X/110 and 200/X/200, both EA and SEA decreased with the increasing density in the middle layer. Though the top layers of 110/X/110 and 200/X/200 can suffer from large external loads, only a slight deformation occurs.

Furthermore, the larger the density of the middle layer, the lower the EA and SEA of the sandwich beam. So, the SEA of 110/X/110 and 200/X/200 are less than those of 52/X/52. Moreover, the arrangement of layers in the core varied from a positive to negative gradient; both EA and SEA increased due to the larger strength of the top layer, which can suffer a larger load. Furthermore, the following layers with low strength can absorb the energy transferred from the top layer. Thus, as shown in Figure 4, the negative gradient core of 200/110/52 has the largest SEA.

### 3.2. Dynamic Load Cases

The sandwich beams with different cores were subjected to the impact of the projectile, and the impact momentum is in the range of 10 to 20 kg·m/s, corresponding to the impact energy in the range of 12.5 to 50 J. And projectile impacted the top layer side of the sandwich-core, which was marked as the impact side. During the tests, the impact force was recorded by an accelerometer, and the corresponding failure modes were also obtained, which were used to analyze the load transfer mechanism between the core layers.

#### 3.2.1. The Impact Force–Time Curves of the Sandwich-Core 52/X/52 

**Figure 5:**
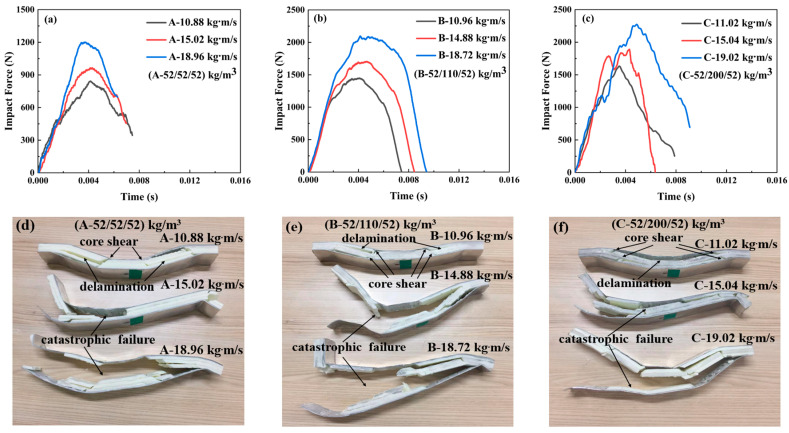
The impact force–time curves and corresponding failure modes of the sandwich beam with different cores: (**a**,**d**) for A-52/52/52; (**b**,**e**) for B-52/110/52; (**c**,**f**) for C-52/200/52.

Figure 5 illustrates that the force curves and failure modes of the sandwich-core 52/X/52 under impact loading differed from the quasi-static load case. The peak force increased with the increasing impact momentum/energy and middle-layer density. In particular, when the density of the middle layer increased from 52 kg/m^3^ to 200 kg/m^3^, the maximum peak forces were 1190 and 2250 N, respectively. It is well known that the maximum shear stress is located at the center of the beam. The increased density in the middle layer can improve the load-sustaining capacity efficiently. Moreover, the density of the top and bottom layers is only 52 kg/m^3^, which is easy to fail due to its low strength. So, the corresponding failure mode is mainly in shear and debonding modes, which is a nearly disintegrated failure mode.

#### 3.2.2. The Impact Force–Time Curves of Sandwich-Core 110/X/110

**Figure 6:**
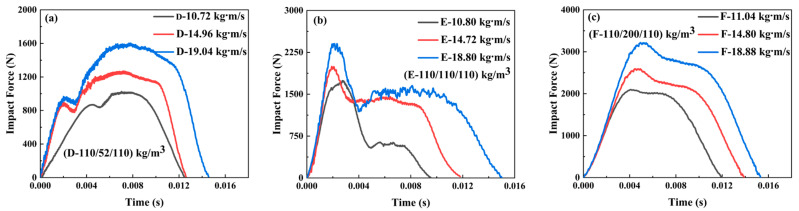

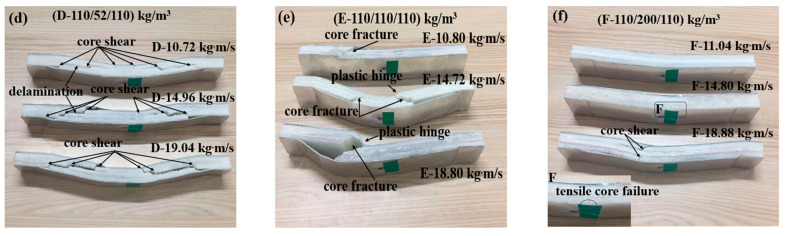
The impact force–time curves and corresponding failure modes of the sandwich beam with different cores: (**a**,**d**) for D-110/52/110; (**b**,**e**) for E-110/110/110; (**c**,**f**) for F-110/200/110.

The variations of the impact force with time of the sandwich beams with sandwich-core 110/X/110 are shown in Figure 6. The impact force increased with the density in the middle layer, which is larger than the results in Figure 5. As the density of the middle layer increased from 52 kg/m^3^ to 200 kg/m^3^, the maximum peak forces were 1570 and 3250 N, respectively. However, the failure modes are pretty different from those in Figure 5; most of the failure modes are in shear failure, and there is nearly no debonding or disintegrated failure. The reason can be explained as follows: the top layer with 110 kg/m^3^ has larger strength and stiffness than that of 52 kg/m^3^, which can sustain a large load and deform in bending deformation, expanding the load exertion area on the next layer. A more effective area of the next layer can be involved in load bearing. So, the impact load of 110/X/110 is larger than that of 52/X/52, and the degree of failure is less than that of 52/X/52.

#### 3.2.3. The Impact Force–Time Curves of Sandwich-Core 200/X/200

**Figure 7:**
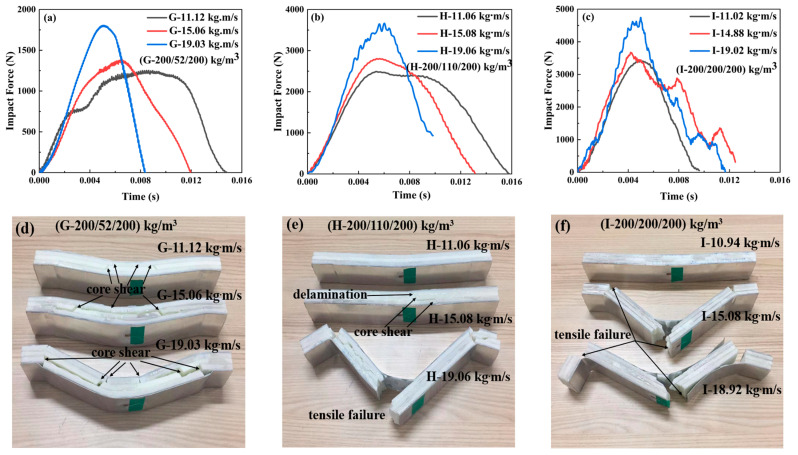
The impact force–time curves and failure modes of the sandwich beam with different cores: (**a**,**d**) G-200/52/200; (**b**,**e**) H-200/110/200; (**c**,**f**) I-200/200/200.

Figure 7 illustrates the impact force curves and failure modes of the sandwich beams with a sandwich-core of 200/X/200. The variation tendency in the impact force of 200/X/200 in Figure 7 was similar to those in Figure 6 and Figure 7. As the density of the middle layer increased from 52 kg/m^3^ to 200 kg/m^3^, the maximum peak forces were 1750 N and 4550 N, respectively. Moreover, it can be observed that the sandwich-core 200/52/200 was in shear failure; however, both 200/110/200 and 200/200/200 failed in tensile failure. The top layer of the 200/X/200 has enough stiffness and strength to suffer the impact of loading and then transfer the load to the middle layer. If the middle layer is 52 kg/m^3^ in density, it would fail in shear failure mode and absorb the energy, which results in a large reduction of the load transferring to the bottom layer, as shown in Figure 7d. However, the middle layer, which is 110 or 200 kg/m^3^ in density, also has enough stiffness and strength, which makes all the layers deform in bending and tensile failure, as shown in Figure 7e,f.

#### 3.2.4. The Impact Force–Time Curves of the Positive and Negative Gradient Cores 52/110/200 and 200/110/52

**Figure 8:**
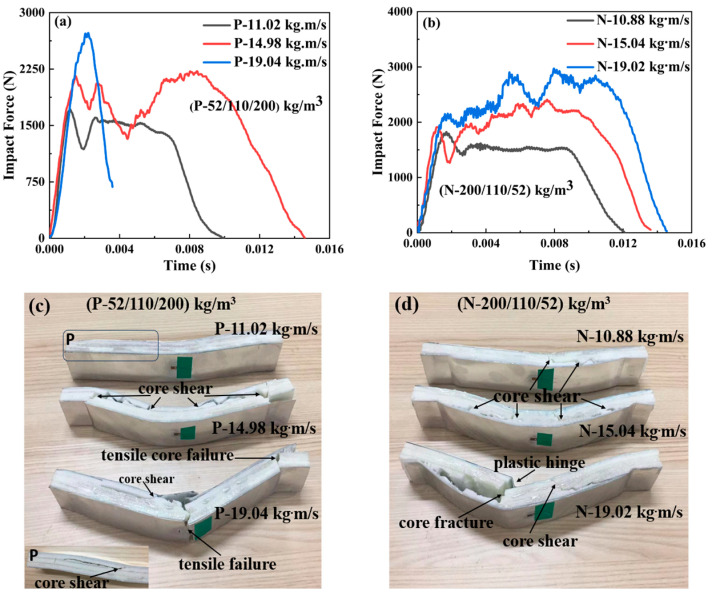
The impact force–time curves and failure modes of the sandwich beams with different cores: (**a**,**c**) positive gradient core (52/110/200); (**b**,**d**) negative gradient core (200/110/52).

The variations of the impact force and failure modes of the sandwich beams with positive and negative gradient cores are shown in Figure 8. For the positive gradient core, the projectile impacted on the top layer core of 52 kg/m^3^, and the bottom layer is 200 kg/m^3^ in density. However, the projectile impacted the top layer core of 200 kg/m^3^ for the negative gradient core, and the bottom layer is 52 kg/m^3^ in density. Both positive and negative gradient cores have a similar variation tendency in the impact force, which increases with the impact momentum/energy. And the impact forces for both cases are at the same level. However, the corresponding duration time of the negative gradient core is larger than that of the positive gradient core at the different impact momentum/energy. It can be observed that the duration time is 0.012 s, 0.014 s, and 0.0145 s for the case of negative gradient core, which is more than 0.009 s, 0.0013 s, and 0.004 s for the positive gradient case, respectively.

Moreover, there is a noticeable difference in the failure modes shown in Figure 8. The failure modes of the positive gradient cores varied from core shear failure to tensile failure; however, all negative gradient cores failed in core shear failure mode. It can be explained as follows: for the negative gradient case, the top layer is 200 kg/m^3^ in density, which can sustain the impact load and then transfer the load to the next layer, but the middle and bottom layers with densities of 110 and 52 kg/m^3^ have low strength, which would fail and collapse under the load transferred from the top layer, and the load and the energy were also dissipated. Thus, the failure mode is a core shear failure. For the positive gradient core, though the top layer of 52 kg/m^3^ failed, the load and energy experienced little loss due to the inertial effect. The concentrated impact load did not spread transversally. Most energy of the impact load still acts on the next layer as a concentrated impact loading, which results in a tensile failure in the bottom layer and face sheet, as shown in Figure 8.

### 3.3. Energy Absorption of the Sandwich Beam with Different Cores under Impact Loading

During calculation of the energy absorption of the sandwich beam with different cores under impact loading, the corresponding force and displacement of the projectile were derived based on the data of the accelerometer, which can also be found in Ref. [47]. The variation in the velocity of the projectile ΔV(t) can be calculated as follows:

(3) $$\Delta V(t)=\int_{0}^{t}a_{0}(t)d(t)$$

Subsequently, the instant velocity of the projectile can be obtained:

(4) $$V(t)=V_{0}-\int_{0}^{t}a_{0}(t)d(t)$$

Hence, the displacement of the projectile during the impact process can be calculated as follows:

(5) $$U(t)=\int_{0}^{t}V(t)d(t)$$

Moreover, the corresponding impact force is as follows:

(6) F(t)=ma(t)

where *a*(*t*), *m*, and *U*(*t*) are the de-acceleration, mass, and displacement of the projectile, respectively; ΔV(t) and *V*(*t*) are the changes in velocity and the initial velocity of the projectile, respectively; *F*(*t*) is the impact force. Then, the corresponding energy absorption can be obtained based on the impact force and displacement using Equations (1)–(3).

**Figure 9:**
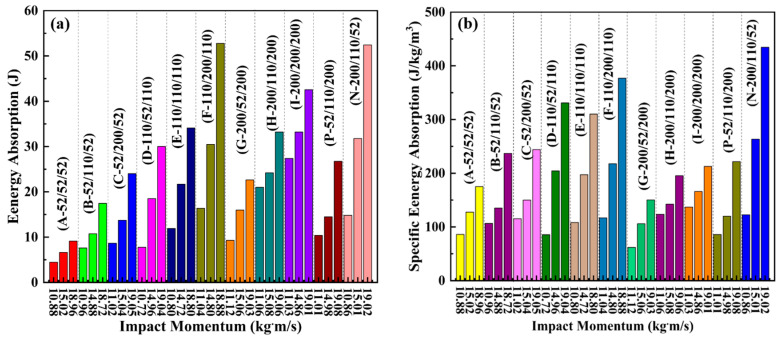
(**a**) Energy absorption and (**b**) specific energy absorption of the sandwich beam with different cores under impact loading.

Based on Equations (1)–(6), the energy absorption (EA) and specific energy absorption (SEA) of the sandwich beams with different cores under impact loading were calculated and shown in Figure 9. Generally, for the sandwich-core, EA increased with the increasing density of the top layer. However, the SEA of 200/X/200 was less than that of 52/X/52 and 110/X/110, which is due to the slight deformation that occurred in the sandwich beam with core 200/X/200, and the sandwich beams with cores 52/X/52 and 110/X/110 absorbed more energy through the deformation and failure of the core.

Furthermore, compared to the sandwich-core, the negative gradient core (200/110/52) has an excellent capacity for both EA and SEA. The top layer of 200 kg/m^3^ can sustain the impact load and transform the concentrated impact load into a distributed load through bending deformation. Then, the following layers absorbed the energy with 110 and 52 kg/m^3^.

### 3.4. Deformation and Failure Mechanism

#### 3.4.1. Deformation and Failure Mechanism of Quasi-Static Load Case

**Figure 10:**
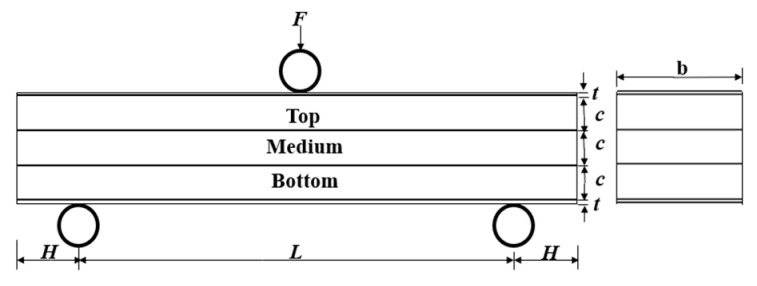
A schematic graph of the sandwich beam with stepwise gradient core under three-point bending.

Most of the failure modes of the sandwich beams are indentation, core shear, and face yield, which can be mapped by the Gibson model [46]. The failure mode map is divided into three regions, each one corresponding to a predominant failure mode, e.g., face yield, indentation, and core shear. And the lines between these failure modes are expressed as follows [48]:

(7) $$\frac{c}{L}=\frac{1}{2}\sqrt{\frac{\sigma_{yc}}{\sigma_{yf}}}-\frac{t}{L}$$

(8) $$\frac{c}{L}=\frac{1}{2}{\left[\left(1+\frac{H}{L}\right)\frac{\tau_{yc}}{\sigma_{yf}}-\frac{2t}{L}\right]}^{-1}{\left(\frac{t}{L}\right)}^{2}$$

(9) $$\frac{c}{L}=\frac{L}{L+H}\frac{\sigma_{yf}}{\tau_{yc}}\left[{\left(\frac{\sigma_{yc}}{\sigma_{yf}}\right)}^{1/2}\frac{t}{L}-\frac{3}{2}{\left(\frac{t}{L}\right)}^{2}\right]$$

where *L* is the effective span length, *b* is the width of the sandwich beam, and *H* is the free end distance outer roller supports. The thickness of the face sheet and each layer foam core are denoted as *t* and *c*, respectively, as shown in Figure 10.

Moreover, the predicted failure load for different modes can be calculated as follows [44]:

The failure load of face yield failure mode is:

(10) $$F_{fy}=\frac{8\sigma_{f}bt(c+t)}{L}+\frac{\sigma_{f}bt^{2}}{L}+\frac{4\sigma_{c}bc(t+c)}{L}$$

The failure load of indentation failure mode is:

(11) $$F_{in}=2bt\sqrt{\sigma_{f}\sigma_{c}}$$

The failure load of core shear failure mode is:

(12) $$F_{cs}=\frac{7bt^{2}}{2L}\sigma_{f}+4\tau_{c}bc\left(1+\frac{2H}{L}\right)$$

In the present paper, the material parameters of the PMI foam are as follows: the yield strength *σ_f_* and elastic modulus *E_f_* of the face sheet are 134 MPa and 68.9 GPa, respectively. And *σ_c_* and *E_c_* are the yield strength and elastic modulus of PMI. For the PMI foam with densities of 52, 110, and 200 kg/m^3^, the yield strength values *σ_c_* are 1 MPa, 3.5 MPa, and 7.5 MPa, and elastic modulus values *E_c_* are 74 MPa, 200 MPa, and 443 MPa, respectively. The shear strength $\tau_{c}\approx \frac{2}{3}\sigma_{Yc}$.

**Figure 11:**
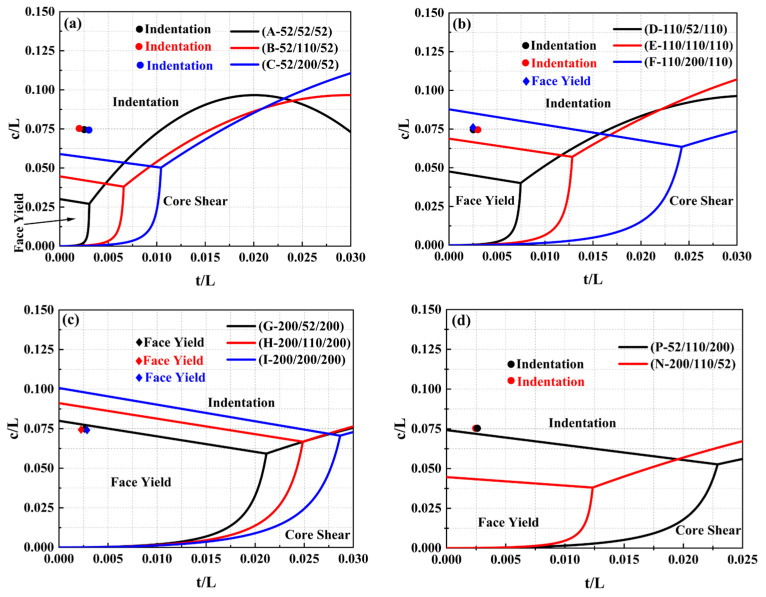
Failure mode map of sandwich beam with different cores: (**a**) 52/X/52; (**b**) 110/X/110; (**c**) 200/X/200; (**d**) 52/110/200 and 200/110/52.

Figure 11 shows the predicted failure mode maps of the sandwich beams with different cores under the three-point bending loading, and the three-point bending experimental results also agreed well with the predicted failure mode map, which demonstrated the reliability of the failure mode map. As shown in Figure 11a–c, when the density of the middle layer of the sandwich-core increased from 52 kg/m^3^ to 200 kg/m^3^, the region of the face yield mode expanded, whereas the region of the indentation and core shear modes shrunk. The reason can be explained by the fact that the middle layer with a large density can improve the stiffness of the sandwich beam and resist the external load. Moreover, as the density of the top layer increased, similar changes in the regions of the failure modes also occurred. Because the top layer with a large density has enough strength and can suffer the load, the sandwich beam would not be in indentation failure. A similar tendency can also be observed in Figure 11d.

#### 3.4.2. Deformation and Failure Mechanisms under Impact Loading

Combined with the impact force curves and failure modes, the deformation and failure mechanisms of the sandwich beam with different cores were analyzed. Generally, the failure modes are sensitive to the layer density (strength), arrangement, and impact momentum/energy, significantly influencing the load-transferring mechanism between the core layers. According to the core type, it can be divided into three cases, including sandwich-core, positive gradient core, and negative gradient core. The details are as follows:

*Case 1: sandwich-core of 52/X/52, 110/X/110, and 200/X/200*

**Figure 12:**
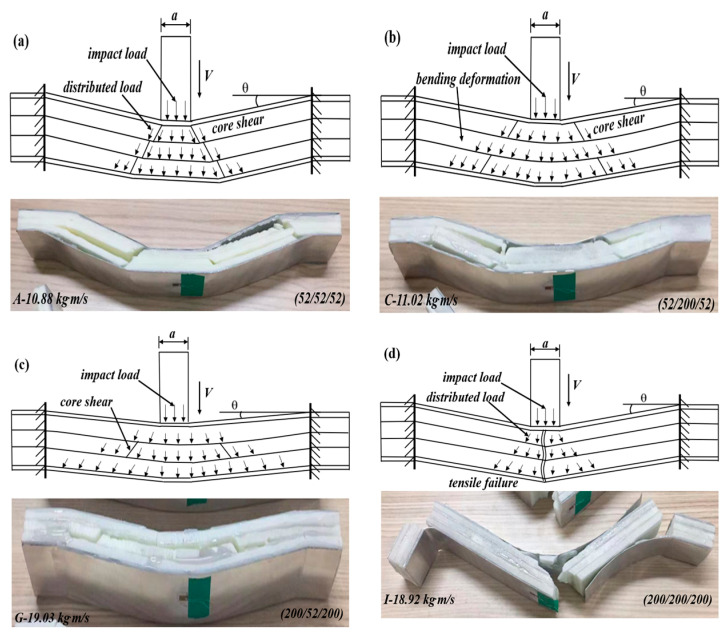
The sandwich beam failure modes and deformation mechanisms with a sandwich-core under different impact momentum: (**a**) specimen of (52/52/52); (**b**) specimen of (52/200/52); (**c**) specimen of (200/52/200); and (**d**) specimen of (200/200/200).

For the sandwich-cores of 52/X/52 and 110/X/110, the strength of the top layer was low, and the top layer failed in shear failure mode under the projectile’s impact. Then, the impact loading would continue to act on the middle layer, but the load exertion area has little change. If the middle layer was still in low density (52 or 110 kg/m^3^), it would also fail in core shear failure and transfer the impact load to the bottom layer, as shown in Figure 12a; if the middle layer was in large density (200 kg/m^3^), it would have enough strength to sustain the load and deform in bending, which can spread the concentrated impact loading into a large area on the bottom layers. Due to the low density of the bottom layer, it would also fail in shear failure mode, as observed in Figure 12b. Still, more energy can be absorbed by its deformation and failure because a more effective part of the bottom layer was involved in energy absorption.

For the sandwich-core of 200/X/200, the top layer can sustain the impact loading and deform in bending, and then the concentrated impact load was distributed and transferred to the middle layer. If the middle layer has a low density, the energy would be absorbed by the collapse deformation and failure of the middle layer, and the load would be further distributed and weakened. When the distributed and weakened load is transferred to the bottom layer, it will deform in bending or tensile failure, as shown in Figure 12c. If the middle layer has a large density, it can sustain the load and deform in bending, further distributing the concentrated load. Still, there is little reduction in energy and load. So, the load applied on the core layers would result in a tensile failure, as shown in Figure 12d.

*Case 2: positive gradient core (52/110/200)*

**Figure 13:**
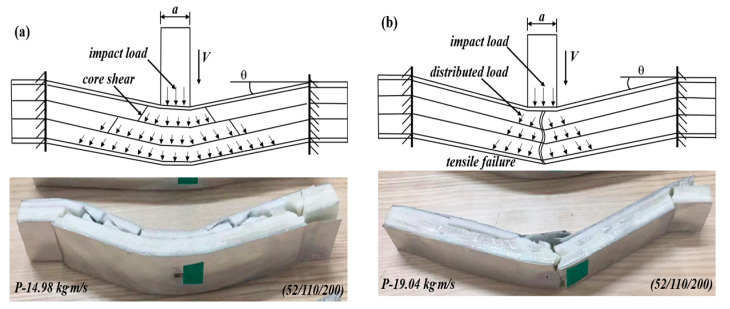
The sandwich beam failure modes and deformation mechanisms with positive stepwise gradient core: (**a**) specimen of (52/110/200); and (**b**) specimen of (200/110/52).

For the positive gradient core of 52/110/200, the top layer of 52 kg/m^3^ failed in shear failure under the impact of the projectile, and the concentrated impact load was distributed and weakened. Then, the load was sustained by the middle layer of 110 kg/m^3^; it would deform in bending and transfer to the bottom layer of 200 kg/m^3^. Thus, both the middle and bottom layers deformed in bending. When the impact momentum/energy is low, the core will fail in shear failure, as shown in Figure 13a. The sandwich beam will fail in tensile failure mode if the momentum is large enough, as observed in Figure 13b.

*Case 3: negative gradient core (200/110/52)*

**Figure 14:**
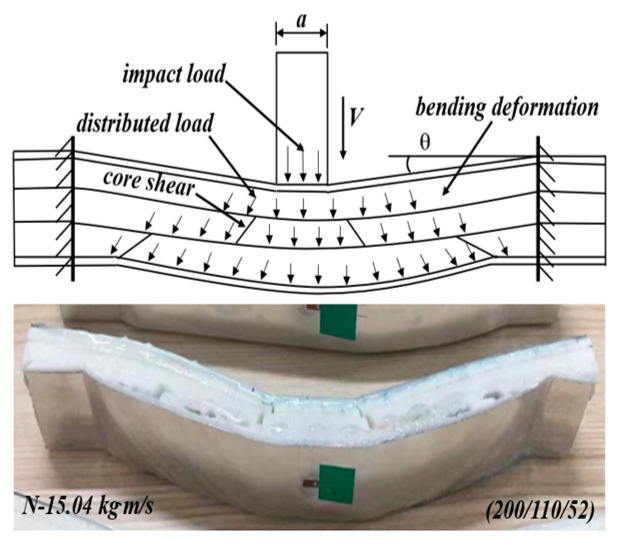
The deformation mechanism of the sandwich beams with a negative gradient core under different impact momentum.

For the negative gradient core of 200/110/52, the top layer has enough strength to sustain the load and deform in bending under the impact load, as shown in Figure 14. Then, the concentrated impact load was also distributed, but the magnitude of the load was a little weakened due to the lower energy absorption. When the distributed load is transferred to the middle layer of 110 kg/m^3^, the middle layer will fail in shear failure mode. The reason is that the strength of 110 kg/m^3^ is much lower than that of 200 kg/m^3^; the middle layer cannot sustain the load in a bending way. After the energy absorption and load spread by the middle layer, the load acted on the 52 kg/m^3^ bottom layer in a large area with a decreased magnitude. Though the 52 kg/m^3^ bottom layer still failed in core shear, most of the energy was absorbed by the middle and bottom layers. The load action area increases layer by layer, and more effective areas of the layers can sustain the load and absorb the energy. The above deformation process and mechanism agreed with the maximum SEA of the sandwich beam with a negative core (200/110/52) in Figure 9.

## 4. Conclusions

The PMI foams with 52, 110, and 200 kg/m^3^ densities were used to design the stepwise gradient cores for the sandwich beams. The dynamic response and failure mechanisms of the different sandwich beams were investigated under three-point bending and impact loading. Combining with the experimental results, the load transferring and failure mechanisms of the sandwich beams were analyzed. The results showed that the arrangement of the layers in the core has a significant influence on the load-bearing capacity and failure modes of the sandwich beam under three-point bending and impact loading. The top layer with a large density can improve the load-bearing capacity, but the middle/bottom layer with a low density promotes the specific energy absorption. For instance, the top layer with low strength (for 52 or 110 kg/m^3^) would fail in indentation (or shear) mode in quasi-static (or impact) load case, respectively, because the top layer cannot dissipate the concentrated load and transfer it to the following layers; however, the top layer with high strength (for 200 kg/m^3^) would deform in bending and transfer the concentrated load into distributed load, which expanded the area to bear the load layer by layer and also improved the energy absorption in each layer. Thus, the 200/110/52 core satisfied the large strength in the top layer and low strength in the middle/bottom layers, resulting in the largest specific energy absorption.

In the future, more work can focus on the performance of stepwise gradient sandwich structures with different materials, such as honeycomb, metal foam, lattice materials, etc. Moreover, different loads can also be considered, such as penetration and blasting loads. The present work provided a valuable method to design an efficient lightweight sandwich structure in the protection fields.

## Figures and Tables

**Table 1:** Details of the sandwich beam specimens.

No.	Specimen Code	Thickness of Each Layer (mm)	Density of the Core Layer (kg/m^3^)	Average Density of the Core (kg/m^3^)
1	A	0.5/5/5/5/0.5	52/52/52	52
2	B	0.5/5/5/5/0.5	52/110/52	71
3	C	0.5/5/5/5/0.5	52/200/52	101
4	D	0.5/5/5/5/0.5	110/52/110	91
5	E	0.5/5/5/5/0.5	110/110/110	110
6	F	0.5/5/5/5/0.5	110/200/110	140
7	G	0.5/5/5/5/0.5	200/52/200	151
8	H	0.5/5/5/5/0.5	200/110/200	170
9	I	0.5/5/5/5/0.5	200/200/200	200
10	P	0.5/5/5/5/0.5	52/110/200	121
11	N	0.5/5/5/5/0.5	200/110/52	121

## Data Availability

The raw data needed to reproduce these findings cannot be shared at this time, as the data will be used in ongoing research.

## References

[1] Li J., Gao G., Yu Y., Zhuo T. (2024). Experimental and numerical study on the lightweight design of load-bearing energy absorption structure for subway train. Thin-Walled Struct..

[2] Ghanbari J., Panirani P.N. (2024). A hybrid bio-inspired sandwich structures for high strain rate energy absorption applications. Sci. Rep..

[3] Xiao W., Hu Y., Li Y. (2023). Ice impact response and energy dissipation characteristics of PVC foam core sandwich plates: Experimental and numerical study. Mar. Struct..

[4] Zhang J., Huang W., Yuan H., Wu X. (2023). Failure behavior of a sandwich beam with GLARE face-sheets and aluminum foam core under three-point bending. Thin-Walled Struct..

[5] Zhao X., Wei L., Wen D., Zhu G., Yu Q., Ma Z.D. (2021). Bending response and energy absorption of sandwich beams with novel auxetic honeycomb core. Eng. Struct..

[6] Khan F., Hossain N., Mim J.J., Rahman S.M., Iqbal M.J., Billah M., Chowdhury M.A. (2024). Advances of Composite Materials in Automobile Applications—A Review. J. Eng. Res..

[7] Zhang J., Huang W., Miao F., Du J., Sun H. (2022). Plastic behavior of sandwich beams with fiber metal laminate face-sheets and metal foam core: Combined local denting and overall deformation. Thin-Walled Struct..

[8] Zhu Y., Sun Y. (2020). Dynamic response of foam core sandwich panel with composite face sheets during low-velocity impact and penetration. Int. J. Impact Eng..

[9] Li Z., Gao Y., Wang Y., Xue P., Gong C., Wang W., Wei X., Xiong J. (2023). Failure mechanisms and acoustic emission pattern recognition of all-CFRP cylindrical honeycomb sandwich shell under three-point bending. Compos. Sci. Technol..

[10] Ge J., Luo T., Qiu J. (2024). Experimental Investigation of the Dynamic Responses of Thin-Walled and Foam-Filled Steel Tubes Subjected to Repeated Impacts. Materials.

[11] Lu H., Wang X., Chen T. (2023). Quasi-static bending response and energy absorption of a novel sandwich beam with a reinforced auxetic core under the fixed boundary at both ends. Thin-Walled Struct..

[12] Xie H., Li W., Fang H., Zhang S., Yang Z., Fang Y., Yu F. (2024). Flexural behavior evaluation of a foam core curved sandwich beam. Compos. Struct..

[13] Zhang W., Li J., Wang Z., Li K., Bai C., Qin Q. (2023). The influence of asymmetric faces on low-velocity impact failure of CFRP/aluminum foam composite sandwich beams. Eng. Struct..

[14] Mei J., Chen Y., Liu Z., Liu J., Huang W. (2024). Experimental and theoretical study on the response of the X-frame CFRP sandwich beam under the local impulsive loading. Int. J. Impact Eng..

[15] Tan Z.H., Luo H., Long W., Han X. (2013). Dynamic response of clamped sandwich beam with aluminium alloy foam core subjected to impact loading. Compos. Part B Eng..

[16] Chen C., Airoldi A., Caporale A., Sala M., Yin G., Xiao J. (2024). Impact response of composite energy absorbers based on foam-filled metallic and polymeric auxetic frames. Compos. Struct..

[17] Acanfora V., Zarrelli M., Riccio A. (2023). Experimental and numerical assessment of the impact behaviour of a composite sandwich panel with a polymeric honeycomb core. Int. J. Impact Eng..

[18] Li J., Zhang W., Wang Z., Wang Q., Wu T., Qin Q. (2023). Dynamic response and failure of CFRP Kagome lattice core sandwich panels subjected to low-velocity impact. Int. J. Impact Eng..

[19] Sun H., Yuan H., Zhang J., Zhang J., Du J., Huang W. (2023). Dynamic response of multilayer sandwich beams with foam-filled trapezoidal corrugated and foam cores under low-velocity impact. J. Eng. Struct..

[20] Koohbor B., Ravindran S., Kidane A. (2021). In situ deformation characterization of density-graded foams in quasi-static and impact loading conditions. Int. J. Impact Eng..

[21] Liu H., Ng B.F. (2022). Dynamic response of density-graded foam subjected to soft impact. Compos. Struct..

[22] Astafurov S.V., Maier G.G., Melnikov E.V., Moskvina V.A., Panchenko M.Y., Astafurova E.G. (2019). The strain-rate dependence of the Hall-Petch effect in two austenitic stainless steels with different stacking fault energies. Mater. Sci. Eng. A.

[23] Liu X., Wang Y., He X., Liu H., Cao S. (2024). Deformation failure mechanism and constitutive model of gradient aluminum foam under impact loading. Compos. Struct..

[24] Yang L., Li X., Yang L., Lu J., Wang Z., Yang J. (2022). Experimental and numerical analysis of dynamic response of graded PVC foam sandwich panel under impact load. Mech. Adv. Mater. Struct..

[25] Kazemi M. (2021). Experimental analysis of sandwich composite beams under three-point bending with an emphasis on the layering effects of foam core. Structures.

[26] Kazemi M. (2022). Experimental investigation on the energy absorption characteristics of sandwich panels with layering of foam core under quasi-static punch loading. J Mech. Adv. Mater. Struct..

[27] Nia A.A., Kazemi M. (2020). Experimental study of ballistic resistance of sandwich targets with aluminum face-sheet and graded foam core. I.J. Sndwich Struct. Mater..

[28] Dhaliwal G.S., Newaz G.M. (2020). Flexural response of degraded polyurethane foam core sandwich beam with initial crack between face sheet and core. Materials.

[29] Wang F. (2021). Static plastic analysis of metallic sandwich beam with functionally graded core. Int. Eur. J. Mech. A/Solids.

[30] Jing L., Su X., Chen D., Yang F., Zhao L. (2019). Experimental and numerical study of sandwich beams with layered-gradient foam cores under low-velocity impact. Thin-Walled Struct..

[31] Zhang W., Qin Q., Li K., Li J., Wang Q. (2021). Effect of stepwise gradient on dynamic failure of composite sandwich beams with metal foam core subject to low-velocity impact. Int. J. Solids Struct..

[32] Fang B., Huang W., Xu H., Jiang C., Liu J. (2022). High-velocity impact resistance of stepwise gradient sandwich beams with metal foam cores. Thin-Walled Struct..

[33] Zhao Z., Jing L. (2020). The response of clamped sandwich panels with layered-gradient aluminum foam cores to foam projectile impact. Mech. Adv. Mater. Struct..

[34] Khaire N., Gupta M., Tiwari G. (2023). Blast resistance of graded aluminium foam core sandwich structure against blast loading. Mater. Today Proc..

[35] Khondabi R., Khodarahmi H., Hosseini R., Ziya-Shamami M. (2023). Dynamic plastic response of sandwich structures with graded polyurethane foam cores and metallic face sheets exposed to uniform blast loading: Experimental study and numerical simulation. J. Braz. Soc. Mech. Sci. Eng..

[36] Flores-Johnson E., Li Q. (2011). Experimental study of the indentation of sandwich panels with carbon fibre-reinforced polymer face sheets and polymeric foam core. Compos. Part B Eng..

[37] Cheon S., Yu S., Kim K.-Y., Lim D.Y., Lee J.-C. (2021). Improvement of Interfacial Bonding Force between PMI Foam and CFRP in PMI Foam-Cored CFRP Sandwich Composites. Fibers Polym..

[38] Huo X., Jiang Z., Luo Q., Li Q., Sun G. (2022). Mechanical characterization and numerical modeling on the yield and fracture behaviors of polymethacrylimide (PMI) foam materials. Int. J. Mech. Sci..

[39] Song S., Xiong C., Zheng J., Yin J., Zou Y., Zhu X. (2021). Compression, bending, energy absorption properties, and failure modes of composite Kagome honeycomb sandwich structure reinforced by PMI foams. Compos. Struct..

[40] Mahgoub M., Zhang Y., Yang C., Tan Z.H. (2023). Dynamic Responses of Sandwich Beams with Polymethacrylimide (PMI) Foam Cores When Subjected to Impact Loading. Materials.

[41] Zhou H., Liu R., Hu Y., Song P., Guo R. (2021). Quasi-static compressive strength of polymethacrylimide foam-filled square carbon fiber reinforced composite honeycombs. J. Sandw. Struct. Mater..

[42] Wang F., Ming Y., Zhao Y., Yang F., Lou J., Zhu Y., Duan Y., Wang B., Xiao H. (2024). Fabrication of a novel continuous fiber 3D printed thermoset all-composite honeycomb sandwich structure with polymethacrylimide foam reinforcement. J. Compos. Commun..

[43] Suzhou Zhongbao Composite Material Co., Ltd., Suzhou, China. http://www.szzbmf.com.

[44] Lutai Co., Ltd., Suzhou, China. http://www.lttc.com.cn.

[45] Kaboglu C., Yu L., Mohagheghian I., Blackman B.R., Kinloch A.J., Dear J.P. (2018). Effects of the core density on the quasi-static flexural and ballistic performance of fibre-composite skin/foam-core sandwich structures. J. Mater. Sci..

[46] Yuan H., Zhang J., Sun H. (2022). The failure behavior of double-layer metal foam sandwich beams under three-point bending. Thin-Walled Struct..

[47] Zhao Y., Yang Z., Yu T., Xin D. (2021). Mechanical properties and energy absorption capabilities of aluminium foam sandwich structure subjected to low-velocity impact. Constr. Build. Mater..

[48] Shunmugasamy V.C., Mansoor B. (2018). Aluminum foam sandwich with density-graded open-cell core: Compressive and flexural response. Mater. Sci. Eng. A.

